# The association between inflammatory markers, walking speed, and metabolic syndrome in older Chinese adults

**DOI:** 10.1007/s40520-025-02984-y

**Published:** 2025-02-27

**Authors:** Dabing Dai, Lican Zhao, Shuai Li, Yu Xu, Aiping Du

**Affiliations:** 1 Department of Critical Care Medicine, West China Hospital, Sichuan University, No.37 Guoxue Lane, Wuhou District, Chengdu, Sichuan Province China; 2 Department of Critical Care Medicine, West China Hospital, West China School of Nursing, No.37 Guoxue Lane, Wuhou District, Chengdu, Sichuan Province China

**Keywords:** Metabolic syndrome, Inflammatory markers, Walking speed, Older adults, Chronic inflammation

## Abstract

**Objective:**

As China’s ageing process accelerates, the annual prevalence of Metabolic Syndrome (MetS) among older Chinese adults continues to rise. This study seeks to assess the relationship between inflammatory markers, walking pace, and MetS in old Chinese adults.

**Methods:**

This study utilised a cross-sectional design, drawing on data from the 2011 and 2015 waves of the China Health and Retirement Longitudinal Study (CHARLS) conducted by Peking University, encompassing 3587 older adults aged over 60. Data regarding inflammatory markers (CRP), walking speed, and variables associated with MetS (including waist circumference and blood pressure) were gathered. Multiple linear regression analysis was used to evaluate the relationship between CRP, walking speed, and MetS.

**Results:**

In a cohort of 3587 older Chinese adults, slower walking speed (β = 0.414) and elevated CRP levels (β = 0.209) were significantly correlated with MetS, with the association persisting after controlling for confounding variables. Furthermore, females, urban residents, individuals with a higher BMI, and smokers exhibited an increased risk of developing MetS.

**Conclusion:**

Walking speed and CRP levels are critical determinants in evaluating the risk of MetS in older adults; improving walking speed and mitigating inflammation may contribute to a decreased risk of MetS.

## Introduction

As the global population ages rapidly, the prevalence of diseases among older adults has generally escalated, rendering elderly health issues a significant global concern [1]. China, as one of the nations with the largest elderly demographic, encounters particularly acute health challenges associated with ageing. Metabolic Syndrome (MetS) encompasses a cluster of metabolic disorders, including hypertension, insulin resistance, obesity, and dyslipidaemia, which markedly elevate the risk of cardiovascular diseases in older adults [2, 3]. The incidence of MetS among the older population is rising worldwide. WHO data indicates a prevalence ranging from 11 to 43%, with 50% in the United States, 27.2% in Italy, 60% in Ecuador, and 18.4% in China [4–8]. Consequently, examining the factors associated with MetS is crucial for enhancing health management among the aged.

Obesity, sedentary behavior, sleep, and physical activity are known risk factors associated with MetS [9]. Walking speed is one of the simplest and most practical methods for assessing the physical function in older adults [10]. Studenski et al. conducted a pooled analysis of data from nine cohort studies involving a total of 34,485 community-dwelling older adults found that for every 0.1 m/s increase in walking speed, survival rate increased by 0.88, indicating that faster walking speed is generally associated with better health in older adults [11]. A cross-sectional study also indicated that increasing daily physical activity, such as the number of steps walked, helps reduce the risk of developing MetS [12]. Furthermore, the 2011–2016 National Health and Nutrition Examination Survey (NHANES) study in the United States showed that for each one-unit increase in the systemic inflammation index (SII), the incidence of MetS increased by 44%, suggesting a significant association between chronic inflammation and MetS [13]. In individuals with MetS, inflammatory markers such as CRP, IL-6, and TNF-α often show an elevated trend. This prolonged low-grade inflammation may expedite the onset of metabolic disorders by influencing insulin sensitivity and lipid metabolism [14].

The impact of socio-cultural and lifestyle factors on this relationship may be distinctive within the older population of China. Nonetheless, studies examining the association between inflammatory markers, gait velocity, and MetS among the older population in China remain inadequate.

This study seeks to investigate the association between inflammatory markers, walking speed, and MetS in the older population of China. The objective is to furnish scientific evidence for the formulation of health interventions aimed at enhancing the well-being of elderly individuals in China and mitigating the risk of MetS.

## Methods

### Study population

The data for this study were sourced from the China Health and Retirement Longitudinal Study (CHARLS) conducted by Peking University, which seeks to gather high-quality microdata on households and individuals aged 45 and older in China. The national baseline survey of CHARLS was executed in 2011, encompassing 150 counties, 450 villages, and approximately 17,000 individuals. These participants are monitored every two to three years subsequently. All participants provided informed consent and received ethical approval from the Peking University Institutional Review Board (IRB00001052-11015).

**Fig. 1:**
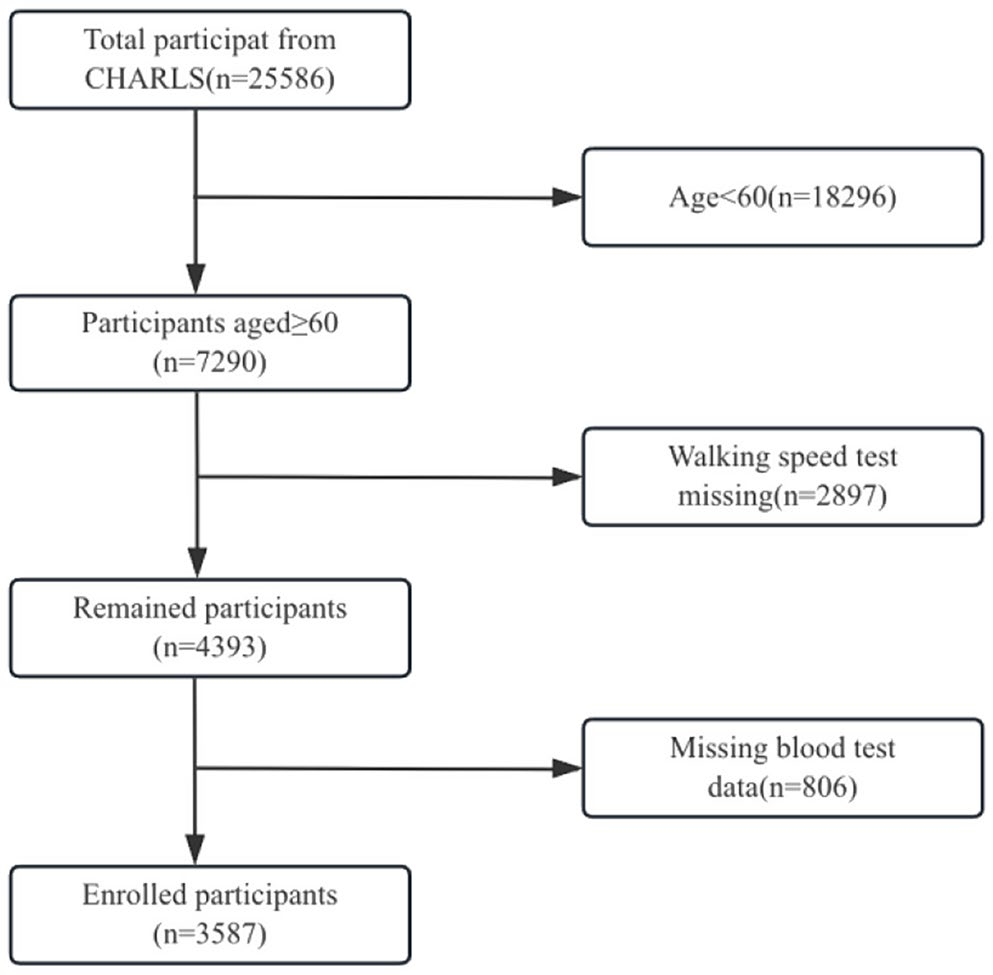
Flowchart for Recruitment of Participants in the China Health and Aging Tracking Survey (CHARLS) 2011

The CHARLS study published blood test data exclusively for 2011 and 2015; consequently, this study utilized data from these two surveys, designating 2011 as the baseline (*n* = 25,586). Participants aged 60 and above were selected (*n* = 7,290). Those with missing key variables or other covariates were excluded (*n* = 3,621). Thus, a total of 3,587 participants were incorporated into this study. Figure 1 illustrates the sample selection flowchart.

### Inflammatory markers

The CHARLS database contains extensive blood test data, with blood samples collected and analyzed from the target population through free health check-ups in 2011 and 2015.The supplementary measurements of the blood test data were strictly conducted according to standard operating procedures and were collected by staff from the Chinese Center for Disease Control and Prevention (CDC).All participants were required to fast for one night before blood collection, and three tubes of blood were collected from each participant.The first tube of blood (2 mL) was used for a complete blood count (CBC) test, covering indicators such as white blood cells, hemoglobin, hematocrit, platelet count, and mean corpuscular volume.The collected venous blood samples were transported at 4 °C to local CDC laboratories or automated analyzers at township-level hospitals near the research sites for analysis.The second tube containing 4 mL of whole blood was used to extract plasma and the buffy coat (white blood cell-rich layer).The blood was separated within the same time frame as the CBC measurement, and if transportation was required, it was sent to local laboratories at 4 °C.The plasma was aliquoted into three 0.5 mL frozen tubes, while the buffy coat was stored in a separate frozen tube.All samples were stored at − 20 °C and transported to the Chinese CDC (Beijing) within two weeks, where they were kept at − 80 °C for further analysis. A 2 mL tube of whole blood was collected for glycated haemoglobin (HbA1c) testing. The blood samples were stored at 4 °C post-collection and subsequently transported to the Chinese CDC (Beijing), where they were ultimately preserved at − 80 °C for analysis. It is significant that high-sensitivity C-reactive protein (CRP), an inflammatory marker, accurately indicates the level of systemic inflammation and has been incorporated into the measurement parameters of this blood test data.

### Assessment of walking speed

All volunteers aged 60 years and older were eligible for the test, and each subject received a safety evaluation. Eligibility for participation was assessed by investigating if recent surgery, injury, or other health concerns had resulted in any walking impairments. In the absence of significant restrictions, the test commenced. Walking speed was evaluated by measuring the duration required to traverse a 2.5-meter distance. The procedure prioritised safety assessments, explicit instructions, and precise timing of two trials, facilitating the quantification of walking speed and the evaluation of participants’ physical activity levels. The mean duration from the two trials was converted into walking speed (m/s). Referring to the results of Liu et al. showed that the walking speed in this study was categorised as high (≥ 0.93 m/s), medium (0.70–0.93 m/s), and low (≤ 0.70 m/s) based on the low 33rd and 66th percentile [10].

### MetS

During the physical examination, all participants underwent general physical measurements and blood tests. The diagnostic criteria for Metabolic Syndrome (MetS) include a waist circumference (WC) of ≥ 80 cm for females or ≥ 90 cm for males, along with any two of the following criteria: (1) Triglycerides (TG) > 150 mg/dL (1.7 mmol/L); (2) High-density lipoprotein (HDL) below the standard (males < 40 mg/dL [1.03 mmol/L], females < 50 mg/dL [1.29 mmol/L]); (3) Systolic blood pressure (SBP) ≥ 130 mmHg or diastolic blood pressure (DBP) ≥ 85 mmHg, or presently utilising antihypertensive medications; (4) Fasting plasma glucose (FPG) ≥ 100 mg/dL (5.6 mmol/L), or the current use of antidiabetic medications.

### Covariates

This study encompassed various potential confounders, including age, gender, education, place of residence, marital status, BMI, self-reported health status, current smoking, alcohol consumption, and chronic comorbidities. Education was classified as (1 = Primary school or below; 2 = Junior high school or above). Place of residence was categorised as (1 = Rural; 2 = Urban). Marital status was defined as (1 = Married; 2 = Single, divorced, or widowed). Smoking status was indicated as (1 = Currently smoking; 2 = Currently not smoking). Alcohol consumption was classified as (1 = Currently drinking; 2 = Currently not drinking). Chronic diseases comprised 14 conditions, including hypertension, diabetes, stroke, lung disease, and kidney disease, and were categorised by the number of conditions as 0, 1, or ≥ 2.

### Statistical analysis

Statistical analysis in this study was performed using R version 4.2.0.Continuous variables are expressed as means and standard deviations, while categorical variables are presented as percentages.Multiple linear regression was used to analyze the relationship between inflammatory factors, walking speed, and MetS.Model 1a: The relationship between walking speed and MetS was analyzed by adjusting for age, gender, education, place of residence, marital status, and BMI.Model 1b: Based on Model 1a, potential confounders such as smoking, alcohol consumption, and chronic comorbidities were further added.Models for inflammatory markers and MetS: Models 2a and 2b are similar to Models 1a and 1b, focusing primarily on the relationship between CRP and MetS.We reported the unstandardized regression coefficients (B) and their corresponding standard errors for all regression analyses.Standardized regression coefficients (β) were also provided to report the study variables measured on different scales.

## Results

A total of 3,857 participants were included in this study, with 3.2% reporting Metabolic Syndrome (MetS). Among those with MetS, the mean age was 66.3 ± 5.5 years, comprising 79.5% male and 20.5% female. The average C-reactive protein (CRP) level was 7.2 ± 10.5, and the mean walking speed was 0.7 ± 0.2 m/s. Within the MetS cohort, 9.0% were classified as having high walking speed, approximately 35.3% as medium, and 55.7% as low. Refer to Table 1 for specifics.

**Table 1:** Basic characteristics related to MetS among Chinese aged over 65 years old (*n* = 3587)

Characteristic	Overall (*N* = 3587)	Non-MetS (*N* = 2439)	MetS (*N* = 1148)	*P*
**Age(mean (SD))**	66.3 (5.9)	66.3 (6.1)	66.3 (5.5)	0.859
**BMI (mean (SD))**	23.2 (12.2)	22.4 (14.5)	24.9 (3.5)	< 0.001
**CRP (mean (SD))**	4.2 (8.8)	2.7 (7.4)	7.2 (10.5)	< 0.001
**Walking_Speed (mean (SD))**	0.9 (0.5)	1.1 (0.6)	0.7 (0.2)	
**Sex (%)**				< 0.001
Female	1347 (37.6)	1112 ( 45.6)	235 ( 20.5)	
Male	2240 (62.4)	1327 ( 54.4)	913 ( 79.5)	
**Residence (%)**				< 0.001
Urban	1204 (33.6)	716 ( 29.4)	488 ( 42.5)	
Rural	2383 (66.4)	1723 ( 70.6)	660 ( 57.5)	
**Marital_status (%)**				0.221
Married	2855 (79.6)	1927 ( 79.0)	928 ( 80.8)	
Unmarried, divorced, and widowed	732 (20.4)	512 ( 21.0)	220 ( 19.2)	
**Education_Status (%)**				0.007
Elementary school or below	3010 (83.9)	2075 ( 85.1)	935 ( 81.4)	
Middle school or above	577 (16.1)	364 ( 14.9)	213 ( 18.6)	
**Smoking_Status (%)**				0.001
Smoker	2145 (59.8)	1504 ( 61.7)	641 ( 55.8)	
Non-smoker	1442 (40.2)	935 ( 38.3)	507 ( 44.2)	
**Drinking_Status (%)**				< 0.001
Drinker	974(27.2)	689( 28.2)	285( 24.8)	
Non-drinker	2613 (72.8)	1750 ( 71.8)	863 ( 75.2)	
**Chronic(%)**				0.509
0	676 (18.8)	407 ( 16.7)	269 ( 23.4)	
1	1053 (29.4)	719 ( 29.5)	334 ( 29.1)	
≥ 2	1858 (51.8)	1313 ( 53.8)	545 ( 47.5)	
**Walking_Speed(%)**				< 0.001
Highest	1165 (32.5)	1062 ( 43.5)	103 ( 9.0)	
Middle	1227 (34.2)	822 ( 33.7)	405 ( 35.3)	
Lowest	1195 (33.3)	555 ( 22.8)	640 ( 55.7)	

Table 2 indicates that Models 1a and 1b demonstrate an association between walking speed and MetS. In Model 1a, reduced walking speed is substantially correlated with MetS (B = 0.410, SE = 0.019, β = 0.414). Moderate walking pace exhibits a positive association with MetS (B = 0.230, SE = 0.018, β = 0.234). Age (B = 0.004, SE = 0.001, β = 0.055), female gender (B = 0.120, SE = 0.016, β = 0.125), and place of residence (B = 0.122, SE = 0.015, β = 0.124) exhibit a positive association with MetS. Model 1b, subsequent to adjustments for BMI, smoking, alcohol intake, and chronic comorbidities, markedly enhanced the model’s explanatory capacity (R² = 21.1%). BMI (B = 0.004, SE = 0.001, β = 0.097), smoking (B = 0.070, SE = 0.017, β = 0.074), and alcohol use are substantially correlated with MetS. Refer to Table 2 for specifics.

**Table 2:** Logistic regression analysis to assess the association between walking speed and MetS

	Model1			
	Model1a		Model1b	
	B(SE)	β	B(SE)	β
Walking_Speed lowest	0.410^***^(0.019)	0.414	0.407^***^(0.019)	0.412
Walking_Speed middle	0.230^***^(0.018)	0.234	0.230^***^(0.017)	0.234
Age	0.004^***^(0.001)	0.055	0.004^***^(0.001)	0.055
Sex_female	0.120^***^(0.016)	0.125	0.180^***^(0.018)	0.187
Residence_urban	0.122^***^(0.015)	0.124	0.111^***^(0.015)	0.112
Marital status_married	0.030^*^(0.018)	0.026	0.030^*^(0.018)	0.026
Education Status_elementary school or below	−0.041(0.020)	−0.032	−0.027(0.020)	−0.022
BMI	-	-	0.004^***^(0.001)	0.097
Smoking Status_smoker	-	-	0.070^***^(0.017)	0.074
Drinking Status_drinker	-	-	0.110(0.028)	0.090
Chronic 0	-	-	0.058(0.019)	0.049
Chronic ≤ 1	-	-	0.013(0.016)	0.012
Observations	3587		3587	
R^2^	0.183		0.211	
F Statistic	114.793^***^		73.338^***^	

Note: *p < 0.1; **p < 0.05; ***p < 0.01

Table 3 illustrates that Models 2a and 2b reveal a significant positive association between CRP and MetS across all models. In Model 2a, CRP exhibits a significant positive association with MetS (β = 0.209, *P* < 0.001). Furthermore, female gender (B = 0.208, SE = 0.016, β = 0.216) and urban residence (B = 0.120, SE = 0.016, β = 0.121) are positively associated with MetS. Model 2b, after adjusting for BMI, smoking, alcohol consumption, and chronic comorbidities, significantly enhanced the model’s explanatory power (R² = 14.7%). Variables such as BMI (B = 0.003, SE = 0.001, β = 0.086), smoking (B = 0.086, SE = 0.018, β = 0.090), and alcohol consumption (B = 0.080, SE = 0.029, β = 0.065) demonstrate certain associations in the model, indicating that these factors may significantly influence the development of MetS. Notably, the impacts of gender and BMI on MetS are more pronounced, with female gender and elevated BMI correlating with an increased risk of MetS. Refer to Table 3 for further details.

**Table 3:** Logistic regression analysis to assess the association between CRP and MetS

	Model2			
	Model2a		Model2b	
	B(SE)	β	B(SE)	β
CRP	0.011^***^(0.001)	0.209	0.011^***^(0.001)	0.209
Age	0.001(0.001)	0.006	0.0004(0.001)	0.006
Sex_female	0.208^***^(0.016)	0.216	0.275^***^(0.018)	0.285
Residence_urban	0.120^***^(0.016)	0.121	0.108^***^(0.016)	0.109
Marital status_married	0.014(0.019)	0.012	0.013(0.019)	0.011
Education status_elementary school or below	−0.031(0.021)	−0.025	−0.017(0.021)	−0.013
BMI	-	-	0.003^***^(0.001)	0.086
Smoking status_smoker	-	-	0.086^***^(0.018)	0.090
Drinking status_drinker	-	-	0.080^***^(0.029)	0.065
Chronic 0	-	-	0.096(0.020)	0.081
Chronic ≤ 1	-	-	0.024(0.017)	0.023
Observations	3587		3587	
R^2^	0.117		0.147	
F Statistic	78.913^***^		51.280^***^	

Note: *p < 0.1; **p < 0.05; ***p < 0.01

## Discussion

This study utilised data from the China Health and Retirement Longitudinal Study (CHARLS) to examine the relationship between inflammatory markers, walking speed, and MetS in older Chinese adults. The findings indicated that elevated CRP levels and reduced walking speed were significantly correlated with MetS. These associations persisted after controlling for confounding variables, offering crucial insights into the risk factors for MetS among the older adults in China.

This study found that slower walking speed is a significant predictor of MetS.Compared to participants with the highest walking speed, those with the lowest walking speed had a significantly increased risk of MetS, and participants with moderate walking speed also showed a certain degree of increased risk.This association remained robust even after adjusting for potential confounding factors such as BMI, smoking, alcohol consumption, and chronic comorbidities (lowest walking speed: B = 0.407, β = 0.412; moderate walking speed: B = 0.230, β = 0.234).Walking speed is a predictive tool for health-related outcomes in the elderly, reflecting their overall muscle health status [15, 16]. It can predict the risk of adverse health events, such as cardiovascular diseases and mortality [17]. A decrease in walking speed may indicate multiple aspects of metabolic dysfunction in older adults.A longitudinal aging study in the UK found that diabetes is a significant risk factor for walking speed decline in tolder adults, with a walking speed decline rate of -0.015 m/s in diabetic patients compared to those without diabetes [18]. A retrospective cohort study in Japan showed that individuals with slower walking speed had an increased risk of cardiovascular disease, which intensified as glucose tolerance impairment worsened [19]. A study by Yamamoto et al. further indicated a significant association between walking speed and metabolic diseases in obese individuals, with faster walking speed improving lipid profiles [20]. Research by Kositsawat et al. showed that IL-6 levels are significantly associated with walking speed in the elderly, with higher inflammation levels closely related to slower walking speed [21]. These mechanisms may interact, accelerating the onset and progression of MetS.This study supports using walking speed as a simple tool for assessing MetS risk in the elderly, and suggests that promoting physical activity and increasing walking speed may effectively reduce the risk of MetS.

Higher CRP levels were significantly associated with MetS, as validated in this study.CRP was significantly positively associated with MetS, and this association remained significant even after adjusting for factors such as BMI, smoking, alcohol consumption, and chronic comorbidities (B = 0.011, β = 0.209).As an important marker of inflammation, CRP is closely associated with chronic low-grade inflammation, which is considered one of the core pathological mechanisms of MetS [22]. Previous studies have shown that chronic inflammation may exacerbate metabolic dysfunction by interfering with insulin signaling, lipid metabolism, and endothelial function [23, 24]. This study further suggests that reducing CRP levels could be an effective strategy for intervening in MetS. Related research has shown that improving dietary patterns, increasing omega-3 fatty acid intake, reducing saturated and trans fats, and increasing the intake of fruits, vegetables, nuts, and whole grains can enhance the body’s anti-inflammatory response, reduce inflammation levels, and thereby reduce the risk of MetS to some extent [25].

This study further examined the influence of demographic characteristics and health behaviours on Metabolic Syndrome (MetS). The findings indicated that elderly men exhibited a lower risk of MetS compared to elderly women, aligning with prior research [26–28]. This disparity may be attributed to men’s lifestyle choices, as they generally engage in higher rates of smoking, alcohol consumption, and betel nut chewing [29]. Additionally, the study identified a significant association between smoking and alcohol consumption and MetS. These findings underscore the detrimental effects of unhealthy lifestyles on metabolic health. Notably, older adults residing in urban areas demonstrated a markedly higher risk of MetS than their rural counterparts, potentially reflecting urbanization-related factors such as sedentary behaviour, high-calorie diets, and psychological stress, which elevate the risk of MetS [30–32]. In addition, the association between BMI level and MetS was also verified in this study, and the use of BMI as a measure of obesity has an important role in predicting metabolic disorders [33]. These results suggest that in the prevention and management of MetS, interventions should be differentiated according to the characteristics of different populations in order to control and reduce MetS more effectively.

## Conclusion

This study identified a significant association between reduced walking speed and elevated CRP levels with MetS, which persisted after controlling for confounding variables. Future research should explore the causal mechanisms linking walking speed and CRP to the onset of MetS, and devise comprehensive intervention strategies aimed at enhancing physical activity and managing inflammation to improve the metabolic health of older adults.

## Limitations

Despite the large sample size and detailed variable analysis in this study, there are still limitations.First, as a cross-sectional study, it cannot establish causal relationships between walking speed, CRP levels, and MetS.Secondly, walking speed and CRP levels may be influenced by unmeasured confounding factors, such as diet and psychological status.Finally, the sample source may limit the generalizability of the findings, and future research will need to validate these results in a broader population.

## Data Availability

CHARLS data have been deposited in the China Longitudinal Study of Health and Retirement database, available at http://charls.pku.edu.cn/. DOI: https://doi.org/10.18170/dvn/wbo7lk.
